# Effect of dietary stevia-based sweetener on body weight and humoral immune response of broiler chickens

**DOI:** 10.14202/vetworld.2021.913-917

**Published:** 2021-04-15

**Authors:** Ramón Miguel Molina-Barrios, Cielo Rubí Avilés-Trejo, María Esthela Puentes-Mercado, Jesús Raymundo Cedillo-Cobián, Juan Francisco Hernández-Chavez

**Affiliations:** Department of Agronomic and Veterinary Sciences, Technological Institute of Sonora, Ciudad Obregon, Sonora, Mexico

**Keywords:** body weight, broilers, immune response, stevia, sweetener

## Abstract

**Background and Aim:**

Steviol glycosides extracted from the leaves of *Stevia rebaudiana* Bertoni have been of much consideration recently because of their beneficial effects on health, raising the possibilities for improving farm animals’ health. Although some studies on stevia’s dietary effect on body weight gain are available, few studies have been conducted to evaluate stevioside supplementation on immune response in broilers. This experiment aimed to analyze how a stevia-based sweetener can affect broiler chickens’ growth performance and humoral response.

**Materials and Methods:**

In this experiment, one hundred and twenty 1-day-old Cobb-line broiler chicks fed with commercial starter/grower diets were included in three groups and supplemented with stevia-based sweetener at levels 0, 80, and 160 ppm, respectively. Chickens were weighed on day 0 and every 7 days for the next 6 weeks. Chicks were then immunized on days 10 and 24 with a Newcastle and infectious bronchitis vaccine and blood sampled on days 7, 24, and 35. Serologic assays were performed to detect specific antibody levels.

**Results:**

The body weight means and body weight gain on day 42 were found to be significantly higher in birds from the group fed with 80 ppm of stevia-based sweetener than those in the control group and slightly higher than those in the group supplemented with 160 ppm of stevia-based sweetener. Likewise, on day 35, antibodies against the Newcastle disease virus were higher in the treatment groups. Immune response to infectious bronchitis virus vaccination was not statistically different among the three groups through the experiment.

**Conclusion:**

Stevia-based sweetener at 80 ppm in commercial-based diets improved body weight gain and immune response in broiler chickens at the market age.

## Introduction

In avian feeding, additives are currently being used to improve broiler chickens’ production performance, within the wide range of nutrients, and due to the increase in both national and international regulations restricting the use of growth-promoting antibiotics. Additive research focuses on natural products such as probiotics, prebiotics, symbiotics, organic acids, enzymes, and, recently, phytogenics. These are herbs, spices, or plant extracts that are known to contain antioxidant, antimicrobial, coccidiostatic, anthelmintic, and immunostimulant properties when added to animals’ diet [1]. Phytogenics commonly used in poultry include oregano (*Origanum vulgare*), rosemary (*Rosmarinus officinalis*), sage (*Salvia officinalis*), thyme (*Thymus vulgaris*), and stevia (*Stevia rebaudiana* Bertoni), plants that improve production efficiency and have an effect on the health of the birds studied [2,3].

Stevia (*S. rebaudiana*, Bertoni) (stevia) is a perennial shrub in the family Asteraceae native to Paraguay and Brazil and is now cultivated in Asia, Europe, Canada, and other countries. Stevia leaves have been determined to contain high levels of sweetener glycosides, such as stevioside and rebaudioside A. As a natural sweetener, stevia and its extracts are widely documented in humans, and several commercial presentations of stevia-based sweeteners are available [4,5].

At present, sweeteners have been used in animal feed for pigs and ruminants to promote feed intake and growth performance [6,7]. Sweeteners, such as sucrose, lactose, and glucose, or high-intensity artificial sweeteners, such as saccharin and neotame, increase feed intake and weight in newly weaned pigs [7]. Several studies indicate that the intestinal tract’s health status modulates dietary stevia supplementation’s functional response [8,9].

Stevioside as a prebiotic power increases the multiplication of beneficial microorganisms such as *Lactobacillus* and *Lactobacillales* and reduces *Clostridiales* and *Clostridia* [9]. However, studies on dietary stevia conducted in chickens remain to be scarce. As previously reported [10], a significant effect of 130 ppm of stevia dietary on the weight gain of broiler chickens has been observed, only in the starter period. No effect of 667 mg stevioside/kg of feed on feed intake, body weight gain, and feed conversion of laying hens and meat-type chickens was reported [11]. Nevertheless, few studies examining the effect of dietary supplementation of stevia-based sweeteners on body weight gain in chickens have already been published, and none was elaborating on its effect on the immune response.

Therefore, this experiment aimed to determine the effects of stevia-based sweetener supplementation on the growth performance and humoral immune response of broiler chickens as an alternative feed additive to improve health status and performance.

## Materials and Methods

### Ethical approval

All experimental procedures were conducted according to the Mexican legislation on animal use for experiments and were granted ethical approval by the Instituto Tecnológico de Sonora bioethics committee.

### Study period and location

The experiment was conducted from February to June 2018 in the Livestock Facility area located in the Instituto Tecnológico de Sonora (ITSON), Ciudad Obregon, Sonora, México). Serological tests and statistical analysis were performed in the Pathology Laboratory of the same institute.

### Animals

One-day-old Cobb-line broiler chickens were obtained from a commercial hatchery and raised in the Instituto Tecnológico de Sonora Livestock Facility Area. Birds were reared in floor pens with water and feed provided *ad libitum*. No antibiotics were applied during the entire experiment.

### Source of stevioside

The source of the stevioside used in this trial was stevia-based sweetener (Svetia, Metco^®^, Mexico), a commercial product containing sucrose, isomalt (2%), steviol glycosides (0.5%), and sucralose (0.08%) per gram.

### Experimental design

Chickens were housed on floor pens, randomly incorporated into six groups (each consisting of 20 chicks), and assigned to three feeding treatments to obtain 2-pen replicates/treatment. From day 7 until day 42, chicks were included in one of the three treatments. In treatment A (control), birds were fed commercial starter/grower diets. Chickens on treatments B and C were fed the commercial diets in which 80 and 160 ppm of stevia-based sweetener were added, respectively. Food and water were supplied *ad libitum* through the experimental period. The rearing temperature was 32°C at day old; it was then gradually reduced until 22°C was reached and maintained until the end of the trial. Chickens had 23 h of light per day for the 1^st^ week of age and thereafter 22 h of light per day. To determine body weight gain, individual weighing was performed on day 1 and consecutively every 7 days for the next 6 weeks. Chickens from all groups were vaccinated at 10 and 24 days of age, through oral drops with one dose of 103 EID 50/mL per bird of a mixed vaccine containing the Newcastle disease virus (NDV) strain LaSota and infectious bronchitis virus (IBV) strains Massachusetts and Connecticut (Sota-Conn-Mas, Pecuarius Laboratorios, Mexico). Blood samples were collected from all bird groups on days 7, 24, and 35; serum was separated from the blood and stored at −20°C until tested.

### Serum samples

Blood samples were collected from the right brachial vein using a hypodermic needle and 3 mL syringe. After collection, samples were immediately chilled and centrifuged at 2000× *g* for 7 min; thereafter, the serum was aliquoted into 1.5 mL tubes (Axygen™ MaxyClear Snaplock Microtubes, Fisher Scientific, Mexico) and stored at −20°C until tested.

### Detection of antibodies against NDV

Hemagglutination inhibition (HI) antibody titers of isolated serums were determined as previously described [12]. Briefly, a 2-fold serial dilution of serum samples (1:2-1:1024) was made in a 96-well microtiter plate serum samples (25 μL) in 25 μL of PBS. Then, four hemagglutinin units of NDV (25 μL) were added to each well, and plates were then incubated for 30 min at 27°C. Finally, 25 mL of 1% (v/v) chicken RBC suspension was added to all wells, and plates were allowed to settle down for 45 min. The reciprocal of the last serum dilution with complete inhibition was determined as the sera’s HI antibody titer [12].

### Detection of antibodies against IBV

Specific antibodies against IBV were detected using a commercial indirect IBV ELISA Kit (IDEXX, Maine, USA), following the manufacturers’ instructions. In brief, serum samples (1:10 diluted) from 20 chickens of each group (at 7, 24, and 35 days of age) were added to the wells, and plates were incubated for 1 h at 37°C and then washed 3 times. Subsequently, 100 μL of anti-chicken IgG conjugated to HRP was added. After 1 h of incubation and 3 times washing of the plates, 100 μL of peroxidase substrate solution was added to each well of the plate; then, incubation time in the dark was for 30 min. Finally, after adding the stop solution, the absorbance was measured at 450 nm (Flock Check program, IDEXX).

### Statistical analysis

Data were analyzed using MedCalc Statistical Software version 19.6.4 (MedCalc Software Ltd, Ostend, Belgium). Chicken’s body weight means were compared through ANOVA and Duncan’s test. HI antibody titer was expressed as log 2 of the highest dilution of complete HI; the means were compared using Student’s t-test [13]. The relative level of antibody against IBV in the sample was determined by calculating the sample to positive (S/P) ratio using the following formula:

S/P = sample (OD) − NCX (OD)/PCX (OD) − NCX (OD), where S/P is the sample to the positive ratio, sample (OD) is the OD of test serum, NCX (OD) is the mean OD of negative control, and PCX (OD) is the mean OD of positive control. The means of the values were then compared using Student’s t-test. p<0.05 was considered statistically significant [14].

## Results

### Effects of stevia-based sweetener dietary supplementation on body weight

The body weight means in the 1^st^ week were found to be similar in the three groups; the chickens’ weights were uniform in all three groups, and no significant difference was noted between the three groups (p>0.05) at the start of the experiment. However, on day 42 of age, the body weight means and body weight gain were significantly higher in birds from Group B fed with 80 ppm of stevia-based sweetener (p<0.05) than those in the control Group A and slightly higher than those in Group C supplemented with 160 ppm of stevia-based sweetener, as shown in Table-1.

**Table-1:** Broiler parameters as influenced by stevia-based sweetener dietary supplementation (means±SEM).

Parameters	Control	Treatment	

		Stevia 80 ppm	Stevia 160 ppm
Initial BW (7 days) (g)	144.56±19.00^a^	152.05±23.56^a^	151.40±19.20^a^
BW (28 days) (g)	1239.33±185.43^a^	1342.50±134.58^a^	1294.87±126.73^a^
BW (42 days) (g)	2190.38±260.19^a^	2302.91±262.97^b^	2199.23±239.23^a^
BWG (g d^−1^)	58.44±0.43^a^	61.45±0.37^b^	58.51±0.51^a^

Broilers were served *ad libitum*. All groups were fed a commercial diet, except that feed in the treatment groups was uniformly supplemented with two different concentrations of stevia (80 and 160 mg/kg of feed). ^a,b^Values in a row with different letters are significantly different (p<0.05). SEM=Standard error of the mean

### Humoral response against NDV

The effect of stevia-based sweetener dietary supplementation on humoral response against NDV, as measured by HI and expressed in log 2 (Table-2), indicates that on day 7 before the experiment started, all chickens in groups were negative to antibodies against NDV. On day 35, the HI titer means were significantly higher in treated Groups B and C (4.99 and 4.18 for 80 and 160 ppm, respectively) than that in the control Group A (1.54). Moreover, the coefficient of variation was found to be significantly reduced in the treatment groups. Although chickens in all groups had an excellent response to first immunization, the Newcastle disease antibody level declined quickly in the control group, while in treatment groups, the group antibody levels were maintained.

**Table-2:** Effect of stevia-based sweetener dietary supplementation on humoral response against Newcastle disease virus, as measured by hemagglutination inhibition and expressed in log 2 (means [CV]).

Sampling time	Control	Treatment	

		Stevia 80 ppm	Stevia 160 ppm
DPS 0 (7 days)	0	0	0
DPS 14 (24 days)	4.138 (45.43)^a^	3.862 (61.63)^ a^	3.759 (58.98)^ a^
DPS 28 (35 days)	1.545 (117.80)^a^	4.989 (23.62)^ b^	4.186 (35.68)^ c^

DPS=Day post-supplementation. ^a,b,c^Values in a row with different letters are significantly different (p<0.05)

Respect the proportion of chickens with protective antibody levels; there was no significant difference among the three groups on day 7. However, a remarkable difference was noted between Group A (control) and Groups B and C (treatments) on day 35; Group A presented just 30% of chickens with protective antibody level, whereas Groups B and C presented 97.2% and 87.2% of birds with protective antibodies against NDV. Furthermore, 55% of the chicks from Group A did not develop detectable antibodies that neutralize the hemagglutination activity of the NDV, although two immunizations.

### Humoral response against IBV

The effect of stevia-based sweetener dietary supplementation on humoral response against the ELISA test’s IBV is shown in Table-3. The antibody titer means expressed in S/P values in each group across time were compared. On day 7, before the experiment of supplementation of the stevia-based supplement started, all titers in chickens from the three groups were deemed low, with a high percentage of chickens with no detectable antibody level against IBV (80%-90%). Through the experiment, the antibody titer means increased similarly in all three groups. No significant differences were detected across time in all experimental groups, neither the S/P values means nor the percent of positive birds (p>0.05).

**Table-3:** Effect of stevia-based sweetener dietary supplementation on humoral response against infectious bronchitis virus as measured by the ELISA test and expressed in S/P values (means [CV]).

Sampling time	Control	Treatment	

		Stevia 80 ppm	Stevia 160 ppm
DPS 0 (7 days)	0.084 (134.68)	0.097 (116.98)	0.108 (212.20)
DPS 14 (21 days)	0.311 (116.14)	0.545 (116.71)	0.342 (121.64)
DPS 28 (35 days)	0.324 (154.63)	0.314 (115.84)	0.319 (96.76)

DPS=Day post-supplementation

## Discussion

In the grower stage, there was evidence that stevia-based supplementation in the diet of chickens has increased the body weight gain, especially for those birds supplemented with 80 ppm of stevia-based sweetener. These parameters differ from the previous studies that referred to no stimuli on feed intake or growth performance in broiler chickens supplemented with stevia leaves of stevioside extracts at variable concentrations [14,15]. Some explanations have been proposed to elucidate the effect of dietary stevia supplementation: The improved body weight gain in broiler chickens could be promoted by feed intake through the hypothalamic neuroactive ligand-receptor interaction and the changed composition of the intestinal microbiota [7]; another is the reduction in the pro-inflammatory response after stimulation of the innate immune response in broiler chickens [14].

One of this work’s objectives was to define an improved response to Newcastle disease and infectious bronchitis vaccination with the stevia-based sweetener supplement. Some experiments have demonstrated the pro-inflammatory response’s stevioside supplementation; serum IgG and IgA levels increase after stevioside dietary supplementation [15]. Daneshyar *et al*. [14] proved that 130 mg/kg of stevioside supplementation can increase body weight and suppress pro-inflammatory response in broiler chickens.

The significant impact on the immune response to the NDV through the dietary supplement of stevia-based sweeteners was in agreement with authors who reported increasing antibody titers in chickens immunized when supplemented with probiotics or prebiotics [16,17]. Besharati *et al*. [18] concluded that stevia alcoholic extract had no significant effect on performance but improved humoral immunity of broilers. The effects reported in this current experiment may have been mediated by promoting the colonization of beneficial microbes, including *Lactobacillus* species that exert dendritic cell activation in the intestinal mucosa [9]. Although there is not a profile of expected titters of antibodies against NDV, according to the OIE [12], HI titers higher than l log24 in vaccinated chickens are considered protective against NDV. A remarkable difference was that at least 60% more of chicken with protective antibody level was found in the treatment groups compared with that in the control group. The inclusion of stevia-based sweeteners in poultry’s feed may be beneficial to chicks as a growth promoter and modulator of the immune system.

## Conclusion

Feeding chickens with a stevia-based supplement benefit body weight gain and enhance the immune response to vaccination. In the grower stage, there was evidence that stevia-based supplementation in the diet of chickens increased the body weight gain when the birds were supplemented with 80 ppm. Likewise, the NDVs immune response was found to be remarkably higher in chickens complemented with stevia-based supplements. No effect on immune response against IBV vaccination was detected. Further studies are necessary to explain the physiologic pathway of the effect of commercial stevia-based supplementation.

## Authors’ Contributions

All authors contributed to the study design and drafted the manuscript. CRA and JRC collected the samples and animals follow-up. CRAT and MEP performed serological tests. RMM and JFH analyzed data, reviewed results, and mainly wrote the manuscript. All authors read and approved the final manuscript.
